# The Pathogenic Properties of a Novel and Conserved Gene Product, KerV, in Proteobacteria

**DOI:** 10.1371/journal.pone.0007167

**Published:** 2009-09-25

**Authors:** Dingding An, Yiorgos Apidianakis, Ana Laura Boechat, Regina L. Baldini, Boyan C. Goumnerov, Laurence G. Rahme

**Affiliations:** 1 Department of Surgery, Massachusetts General Hospital and Harvard Medical School, Boston, Massachusetts, United States of America; 2 Shriners Research Institute, Boston, Massachusetts, United States of America; 3 Department of Microbiology and Molecular Genetics, Harvard Medical School, Boston, Massachusetts, United States of America; 4 Departamento de Bioquímica, Instituto de Química, Universidade de São Paulo, São Paulo, Brazil; Baylor College of Medicine, United States of America

## Abstract

Identification of novel virulence factors is essential for understanding bacterial pathogenesis and designing antibacterial strategies. In this study, we uncover such a factor, termed KerV, in Proteobacteria. Experiments carried out in a variety of eukaryotic host infection models revealed that the virulence of a *Pseudomonas aeruginosa kerV* null mutant was compromised when it interacted with amoebae, plants, flies, and mice. Bioinformatics analyses indicated that KerV is a hypothetical methyltransferase and is well-conserved across numerous Proteobacteria, including both well-known and emerging pathogens (e.g., virulent *Burkholderia*, *Escherichia*, *Shigella*, *Vibrio*, *Salmonella*, *Yersinia* and *Brucella* species). Furthermore, among the 197 *kerV* orthologs analyzed in this study, about 89% reside in a defined genomic neighborhood, which also possesses essential DNA replication and repair genes and detoxification gene. Finally, infection of *Drosophila melanogaster* with null mutants demonstrated that KerV orthologs are also crucial in *Vibrio cholerae* and *Yersinia pseudotuberculosis* pathogenesis. Our findings suggested that KerV has a novel and broad significance as a virulence factor in pathogenic Proteobacteria and it might serve as a new target for antibiotic drug design.

## Introduction

Important infection mechanisms are often shared across diverse bacterial pathogens [1], [2], [3], [4], [5], [6]. Identification and understanding of these conserved themes will not only expand our knowledge of specific virulence mechanisms, but will also provide information about the evolution of microbial pathogenesis. A convergence of such findings is also needed to inform new strategies against bacterial infections with wide clinical applications, and to provide new solutions to the ever-growing problem of antibiotic resistance. However, discovering broadly conserved virulence factors faces great challenges caused by the practical limitation mammalian hosts pose in high-throughput approaches [7]. This limitation has been considerably circumvented following the discovery that important virulence factors and corresponding pathways are conserved across a spectrum of hosts ranging from amoebae to mice [2], [8], [9], [10], [11]. This conservation made non-vertebrates amenable surrogate hosts for studying mammalian pathogenesis and added the benefit of enabling broadly conserved virulence factors to be identified. Indeed yeasts [12], plants [13], nematodes [14], fruit flies [15], and zebrafish [16] have all been successfully applied in pathogenesis experiments.

*P. aeruginosa* is used as a model for bacterial pathogenesis study because of its potency as a multi-host pathogen and the abundance of tools that are compatible with it [13], [17], [18]. The present report describes the discovery of a conserved *P. aeruginosa* virulence determinant, KerV. We further report experiments examining the breadth of KerV as a conserved virulence factor, not only against multiple hosts but also in several pathogens.

## Results

### *P. aeruginosa* KerV-mediated virulence is conserved against a spectrum of eukaryotic hosts

In a screen for novel evolutionarily conserved *P. aeruginosa* virulence factors using an *Arabidopsis* infiltration model [13], [19], we identified a mutant with a Tn*phoA* transposon insertion at gene PA14_41070 that exhibited decreased virulence compared to the parental strain PA14. This gene, annotated here as *kerV*, encodes a hypothetical protein with 253 amino acids in length.

**Figure 1:**
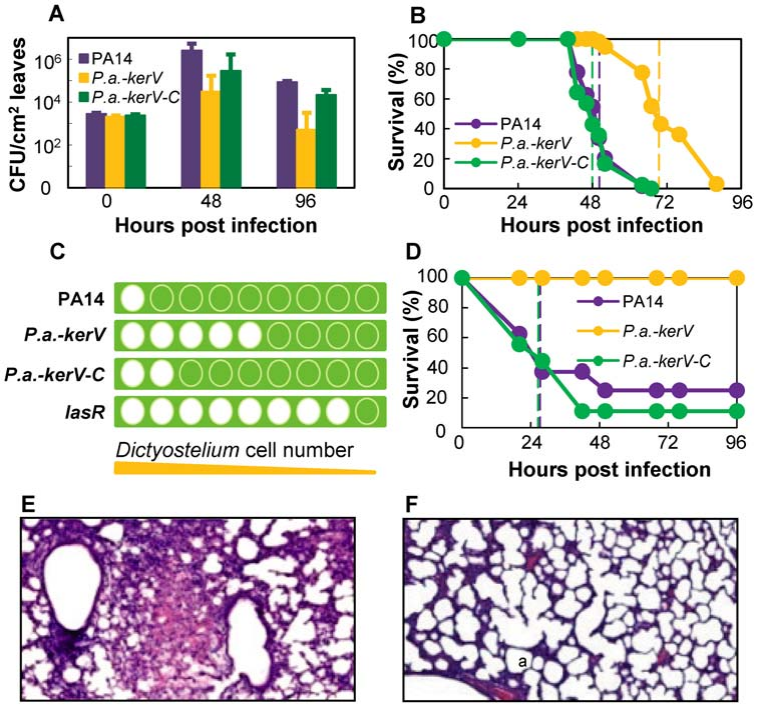
KerV acts as a virulence determinant in *P. aeruginosa* against a range of eukaryotic hosts. (A) Bacteria quantities recovered from infected plant leaves. (B) Fly survival kinetics in a pricking infection model. Dotted lines are color-coded and represent survival medians for corresponding strains. (C) Amoeba phagocytosis of *P. aeruginosa*. The graphic was produced from representative experimental observations. For detailed data see Table S1. Green square, bacterial lawn; green-filled circle, no phagocytosis of bacteria; white-filled circle, clear zone due to phagocytosis of bacteria. From left to right the numbers of laid *Dictyostelium* were 2.0×10^5^, 8.0×10^4^, 3.2×10^4^, 1.3×10^4^, 5.1×10^3^, 2.0×10^3^, 8.2×10^2^, 3.3×10^2^ and 1.3×10^2^. (D) Mouse survival kinetics in an acute lung infection model. (E and F) Histology of the infected neonatal mice lungs by PA14 (E) and *P.a.-kerV* (F) in the acute lung infection model. a-alveoli.

To study the function of KerV in pathogenesis, we constructed and applied a clean in-frame deletion mutant (*P.a.-kerV*) and a gene-replacement complementation strain (*P.a.-kerV-C*) in further analyses. The mutant did not exhibit growth defects compared to the parental strain PA14 in either rich (Luria Bertani broth (LB)) or minimal medium (M9 supplemented with 0.4% glucose) (Figure S1). The ability of *P.a.-kerV* to proliferate within *Arabidopsis* leaves and cause disease symptoms was assessed in the *P. aeruginosa Arabidopsis* infiltration model, which involves forced insertion of suspended bacterial cells into the intercellular space of *Arabidopsis* leaves. At 48 h and 96 h post-infection, the densities of *P.a.-kerV* colony forming units (CFUs) in infected leaves were about two orders of magnitude less than those of PA14 (about 10^4.5^ vs. 10^6.4^ CFU/ml at 48 h; about 10^2.7^ vs. 10^4.9^ CFU/ml at 96 h; P<0.02); meanwhile the densities of CFUs of the complemented strain *P.a.-kerV-C* were similar to those of PA14 (P>0.1, Figure 1A). Accordingly the intensity of observed infection symptoms was reduced in leaves infected with the *P.a.-kerV* mutant, but restored in leaves infected with *P.a.-kerV-C* (data not shown).

The virulence of these strains was then examined in a *Drosophila* pricking model at 21°C in which about 100 bacterial cells were inoculated into the dorsal thorax of host flies. Flies infected with *P.a.-kerV* survived longer (n = 43, median survival 70 h) than those infected with PA14 (n = 47, 50 h) or *P.a.-kerV-C* (n = 42, 48 h) (P<0.0001, Figure 1B). These results are in agreement with our previous study [20], in which a *kerV* mutant (termed D12) was unable to evade host defense mechanisms in flies and therefore was deemed not proficient in infection.

Given the demonstrated involvement of KerV in both plant and fly pathogenesis, we asked whether KerV is indeed a key *P. aeruginosa* virulence factor important for infection of a broad range of hosts. The mutant's virulence was tested in two additional eukaryotic hosts: amoeba and mouse. In the amoeba phagocytosis assay [21], *Dictyostelium discoideum* with 2.5-fold sequential dilutions starting with 2.0×10^5^ cells were spotted on different *P. aeruginosa* lawns and the number of clear zones that *D. discoideum* made by phagocytosing *P. aeruginosa* was recorded for each bacterial strain. In this assay, greater virulence is associated with a smaller number of clear zones and a bigger number of minimally required *D. discoideum* cells. Indeed, there was only one clear zone on the representative lawn of virulent PA14, indicating a minimum of 2.0×10^5^ *D. discoideum* cells were needed for phagocytosis of PA14 (Figure 1C and Table S1). In contrast, eight clear zones (corresponding to about 3.3×10^2^ *D. discoideum* cells) were shown on a representative lawn of the negative control *lasR* (a strain deficient in producing the master virulence regulator LasR [10]). Meanwhile, five clear zones (corresponding to about 5.1×10^3^ *D. discoideum* cells) were shown on a representative *P. a.-kerV* lawn and two clear zones (corresponding to about 8.0×10^4^ *D. discoideum* cells) on a representative *P.a.-kerV-C* lawn. Therefore our findings indicate that KerV plays an important role in *P. aeruginosa* resistance to amoeba phagocytosis.

In an acute mouse pulmonary infection model [22] in which 10^7^ bacteria were administered intranasally, *P.a.-kerV* failed to confer any mortality at 96 h post-infection, while the median survival of PA14 and *P.a.-kerV-C* infected mice were both 28 h (P<0.003, Figure 1D). Histopathology of neonatal mouse lung tissues at 24 h post-infection showed that PA14 (Figure 1E) caused a typical lobar pneumonia characterized by pulmonary consolidation and intra-alveolar hemorrhage with massive inflammatory infiltrates. In contrast, neither inflammatory response nor hemorrhage was present inside the alveolar spaces of *P.a.-kerV* infected tissues (Figure 1F). These observations demonstrated that the virulence of the *kerV* mutant was attenuated in the mouse lung infection model and thus aroused much less inflammation. Interestingly, in a burn-mouse model [23], the mutant exhibited similar virulence compared to that of the parental strain in a 5-day experiment using 1×10^5^ bacteria/mouse (P>0.05, Figure S2).

### KerV is a hypothetical methyltransferase and is conserved in a defined genomic region in many Proteobacteria

A homology search in KEGG [24] revealed that KerV has putative methyltransferase type_11 motifs (Figure S3) and is annotated as a S-adenosylmethionine-depended methyltransferase (SAM-MT). SAM-MTs usually have three conserved amino acid motifs for SAM binding [25]. In KerV, the best match for motif I, usually the most conserved and most critical for binding SAM [26], is HAELPPSTG; however, the H and the first P are not present in other known methyltransferases to our knowledge. Secondary structure prediction by PROF algorithm [27] at PredictProtein site (http://www.predictprotein.org/) suggests it also lacks a classic seven-stranded β sheet [26]. Nevertheless, KerV possesses a fine post-I motif (LPGVD), a motif II (ADVVL), and a motif III (TVRPGGHLLL) with a commonly seen spacing of 11 residues between the putative motif I and post-I. In addition, the expected β strand for motif III is also suggested by PROF algorithm. These characteristics suggest that KerV encodes for either a novel methyltransferase with unique features or a protein with little familiarity.

**Figure 2:**
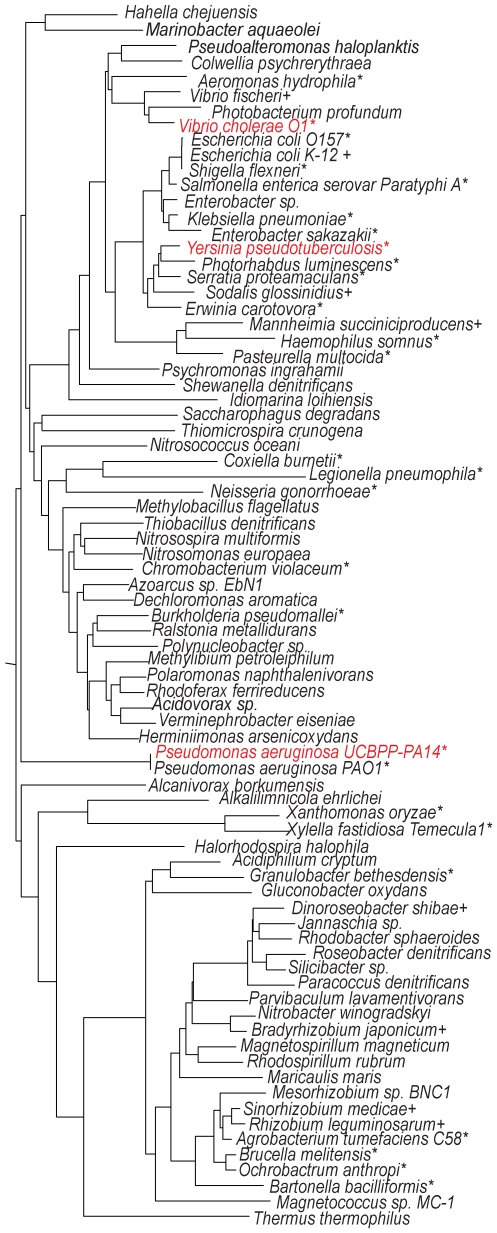
KerV is conserved in Proteobacteria. Phylogenetic tree generated based on selected KerV orthologs using MAFFT in KEGG. The selection criteria were: 1) orthologs with a SW-score over 100; and 2) no more than two representative isolates per genus. * indicates that strains have known pathogenic interactions with eukaryotic hosts; + indicates that strains are known to interact with eukaryotic hosts, but have no known virulence properties. Strains without a label belong to environmental species and do not have substantial known associations with eukaryotes.

A KerV ortholog search in KEGG returned 196 hits spanning mostly β- and γ-Proteobacteria (Smith-Waterman (SW)-score >100 and best-best search, Figure 2 and Table S2), most of which also possess the methyltransferase type_11 motif. These hits include well-characterized and emerging pathogens of humans, plants, insects and nematodes. Among the list are also bacterial strains that are known to have non-pathogenic interactions with hosts, including symbiotic and commensal associations (e.g., certain *Vibrio*, *Enterobacter*, *Sinorhizobium* strains), and environmental species (e.g., *Methylobacillus*, *Nitrosospira*, *Chromobacterium*).

**Figure 3:**
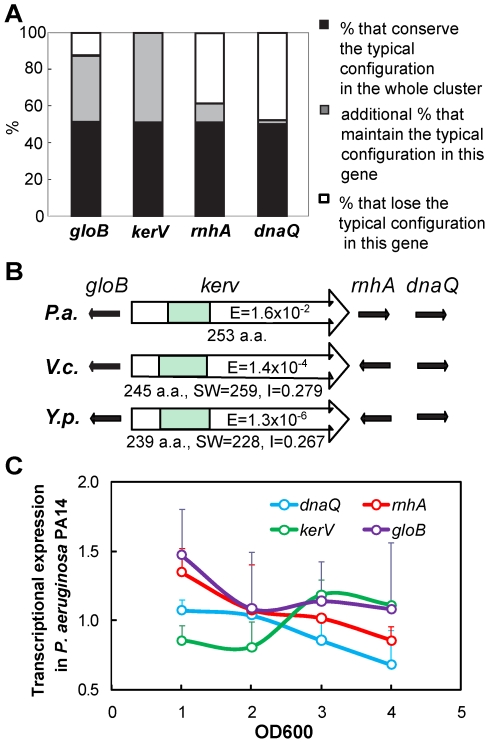
KerV orthologs are located in a conserved genomic neighborhood with typical configuration in many species. (A) Conservation of the typical configuration. Data are based on the 197 bacterial strains that have a KerV ortholog with SW-score >100. (B) The typical configuration in *P. aeruginosa* (*P.a.*), *V. cholerae* (*V.c.*) and *Y. pseudotuberculosis* (*Y.p.*). Green boxes represent the methyltransferase type_11 motif (Pfam08241) and are shown to scale with respective KerV orthologs. E, expectation value for homology with Pfam08241. SW, SW-score of ortholog if searched using PA14 KerV; I, identity of the ortholog. Arrows indicate the predicted transcriptional direction of each gene. (C) Transcriptional expression of *kerV* in PA14 is distinctive from that of *gloB*, *rnhA* and *dnaQ*. Error bars show standard deviations in one experiment. The illustrated trends are representative of multiple experiments.

Examination of *kerV* and orthologs' genomic context by KEGG [28] revealed that they are typically located within a defined neighborhood. In all 197 cases analyzed, 171 (87%) and 120 (61%) of the KerV orthologs appear next to a putative hydroxyacylglutathione hydrolase ortholog (*gloB*, PA14_41080) and a ribonuclease H ortholog (*rnhA*, PA14_41060), respectively. Furthermore, *dnaQ* (PA14_41050), which encodes the ε subunit of DNA polymerase III, often co-localizes with *kerV*. In fact, 100 (51%) of the 197 genomes maintain the exact order of gene orthologs as *gloB-kerV-rnhA-dnaQ*, referred to as the “typical configuration” in this study (Figure 3A and 3B). In addition, 176 genomes (89%) share this conserved region. In these genomes, at least one of the *gloB* and *rnhA* orthologs is maintained in the typical configuration with respect to the *kerV* ortholog and the remaining orthologs are present, if not nearby. A broader less-conserved area was observed beyond the immediate *kerV*-containing region discussed above, but it is not the focus in this study.

GloB, also annotated as glyoxalase II, is a component of the glyoxalase system. It is primarily involved in detoxification of endogenously formed reactive 2-oxoaldehyde species in various processes (e.g. glycolysis) in both prokaryotic and eukaryotic organisms [29], [30]. RnhA specifically degrades RNA in DNA-RNA hybrids and is a key player in DNA replication [31], [32]. *Escherichia coli* RnhA has been described as essential for growth [33]. DnaQ has 3′–5′ exonuclease activity that fixes DNA replication errors. Mutation of this gene can lead to a mutator phenotype [34]. Nevertheless, the mutation frequency of *P.a.-kerV* was verified to be similar to that of the wild-type (data not shown).

We tested a *P. aeruginosa gloB* mutant, retrieved from the PA14 transposon library [18], for virulence in a *Drosophila* pricking model. This mutant exhibited the parental virulence phenotype (data not shown). It is noteworthy that *rnhA* and *dnaQ* mutants are not available in both the PA14 and the PAO1 (another commonly used parental strain) transposon libraries [18], [35], which strongly suggests the indispensability of these two genes for *P. aeruginosa* survival. Furthermore, although oriented in the same direction in the chromosome, the dissimilar gene expression profiles of *kerV*, *rnhA* and *dnaQ* during PA14 growth suggest that they may not be components of a shared operon in the *P. aeruginosa* genome (Figure 3C). The genes *gloB*, *rnhA* and *dnaQ* were relatively actively transcribed during early log phase but this transcription subsequently subsided. On the contrary, *kerV* transcription was found to be up-regulated as cells were approaching the late log phase and entering the early stationary phase. Nonetheless, the close and conserved physical association of *kerV* with these essential genes hints that the virulence mechanism of KerV may be related to fundamental bacterial physiology.

### KerV orthologs mediate pathogenesis in other Proteobacteria

The presence of KerV orthologs in defined genomic context in other important pathogens suggested that KerV might represent a type of generic virulence determinant. We tested this hypothesis in a *Drosophila* feeding infection model employing *kerV* ortholog knockout mutants in two γ-Proteobacteria pathogens: *V. cholerae* and *Y. pseudotuberculosis*.

**Figure 4:**
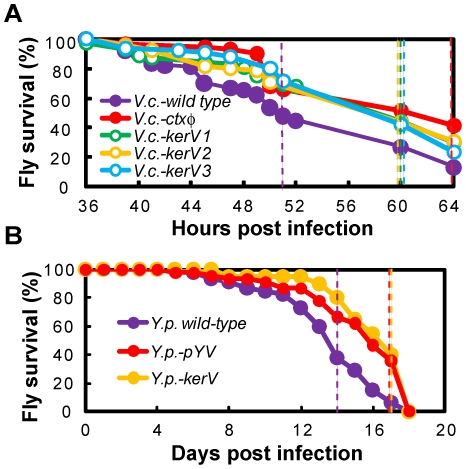
KerV is a conserved virulence determinant in various Proteobacteria. Survival of flies in *Drosophila* feeding infection assays using wild-type and *kerV* mutants in *V. cholerae* (A) and *Y. pseudotuberculosis* (B) background. Known virulence-compromised strain was used in each assay as negative control and the data are presented in red. Dotted lines represent survival medians for the color-corresponding strains. All mutants were tested for growth in LB broth and none had any notable differences from the parental strain.

*V. cholerae*, the aetiological agent of cholera, generally carries two major virulence factors encoded on a lysogenic filamentous bacteriophage (CTXΦ): cholerae toxin (CT) and a toxin co-regulated pilus (TCP) [36]. To test whether KerV ortholog is important for virulence in *V. cholerae*, three unique insertion mutants, labeled *V.c.-kerV1, 2* and *3*, and the wild-type *V. cholerae* were retrieved from a *V. cholerae* transposon library [37] and fed to *Drosophila* to mimic the human disease cholera [38]. This library used a clinical isolate, C6706, which is a pandemic O1 El Tor isolate, as the wild-type. The KerV ortholog in *V. cholerae* O1 is encoded by VC2235 (Table S2). A *ctxφ* mutant in the same parental background was also retrieved and used as a control for attenuated virulence. As expected, the flies infected with the *ctxφ* mutant had higher viability, with a median survival time of 64 h (n = 87) compared to 51 h (n = 91, P<0.0001) for flies infected with the parental strain (Figure 4A). Interestingly, all three *kerV* mutants exhibited a defect comparable to that of the *ctxφ* mutant, yielding a median survival time of 60 h (n≥65, P<0.005). Since the *kerV* ortholog and its adjacent genes are transcribed in opposite directions in *V. cholerae* (Figure 3B), the insertion of a transposon should not cause a polarity effect. Hence the phenotype observed for *V.c.-kerV* mutants can be attributed solely to disruption of the *kerV* ortholog. We therefore conclude that the KerV ortholog is important for *V. cholerae* infection.

*Y. pseudotuberculosis* is a gastrointestinal pathogen with several essential virulence factors on a 70-kb plasmid (pYV) [39]. In the virulent *Y. pseudotuberculosis* parental strain YPIII, KerV ortholog is encoded by YPK_1107, which also does not form an operon with adjacent genes (Figure 3B). The *kerV* deletion mutant, *Y.p.-kerV*, was constructed and tested in an adapted *Drosophila* feeding assay. The assay was a slow killing model in which flies infected with the virulent YPIII had a median survival of 14 days (n = 45, Figure 4B). Flies infected with a control mutant lacking the pYV virulence plasmid, termed *Y.p.-pYV* in this study (YPIII pIB1^−^ in [40]), exhibited slower mortality kinetics with a median survival of 17 days (n = 45, P<0.0001). The *Y.p.-kerV* strain behaved essentially the same as *Y.p.-pYV* with the same median survival time (n = 40, P<0.0001). Our findings provide evidence that the KerV ortholog is a new virulence factor for *Y. pseudotuberculosis*.

## Discussion

In this study, we applied a combined approach of bench experiments and bioinformatics analyses to identify novel virulence determinants. We discovered such a factor KerV in *P. aeruginosa*, *V. cholera*, and *Y. pseudotuberculosis*. Our results strongly suggest that KerV orthologs may be universal virulence determinants since they are conserved in many pathogenic Proteobacteria. The finding that KerV is important for *P. aeruginosa* infection of a broad range of hosts from amoebae to mice further strengthens this identity. The conservation of KerV in non-pathogenic bacteria also proposes that it is likely important in other ecological settings besides plant and animal infections, such as beneficial bacteria-host interactions and environmental processes. Interestingly, KerV orthologs are mostly well-conserved in β- and γ-Proteobacteria with a few exceptions in α-Proteobacteria by our criteria. The exclusiveness of KerV orthologs in Proteobacteria indicates that appearance of KerV is a late event in evolution and is unique to the physiology of this phylum. These analyses indicate that the natural reservoirs of most of the species that have KerV orthologs are not extreme in terms of temperature, pressure, oxygen and other nutrient levels, where higher organisms are easily found. In fact, a lot of these species are known to intimately associate with plants and animals symbiotically or pathogenically, although some have no known associations with eukaryotes. It is very likely that the essential function of KerV is for bacteria to interact with their environments, especially with other cohabitating organisms.

Co-localization of KerV with essential DNA replication and repair genes and reactive species-detoxification gene implies that its pro-virulence effect may not necessarily be related to a direct toxic enzymatic activity, but rather be attributed to a more generic role in fundamental bacterial physiology. Another hint may come from the distinct phenotypes of *P.a.-kerV* mutant in an acute mouse pulmonary infection model and a burn-mouse model, where the mutant was defective in the former model while as fit as the wild-type in the latter. Among the many dissimilarities between the two mouse models, different local host immune efficiency [41] and bacterial nutritional environments (rich environment with readily abundant supplies of proteins, amino acids, polysaccharides etc. at the burn site) are particularly notable. It is possible that KerV function is important for the bacterium to first establish a niche in the lung tissues, where nutrients are not as easily available and a competent host response is equipped, before launching its various known virulence attacks. Indeed, mechanisms involved in the maintenance of normal cell physiology can have profound pathogenic influences [42], [43], [44]. Irrespective of the details, KerV likely represents a not-yet-described virulence pathway. To our knowledge, KerV has never been linked to any known *P. aeruginosa* virulence factors or their regulation systems, such as the las, rhl and mvfR quorum sensing systems, the GacS/GacA/rsmZ pathway, VfR, or the type III secretion system. A preliminary search of published transcriptional and proteomic studies of these systems did not reveal any associations with KerV. However, KerV may be involved with these systems post-transcriptionally or indirectly. Alternatively, KerV's connection can be prominent in settings other than those employed in these studies (e.g., *in vivo* conditions).

KerV's characteristics make it a promising anti-infective target for novel drug development. The fact that the *kerV* null mutants did not exhibit growth defects in any of the three pathogens examined suggests that KerV is not specifically involved in bacterial replication and division. Targeting KerV pharmacologically may provide a strategy to impede virulence without directly interfering with bacterial cell viability. This strategy is therefore critically distinctive from traditional antibiotics as it should greatly reduce selection for drug resistance. Given the significance and conservation of KerV, such an innovation would have broad applications.

## Materials and Methods

### Ethics statement

All animals were handled in strict accordance with good animal practice as defined by the relevant national and local animal welfare bodies. All animal work was approved by the Massachusetts General Hospital Institutional Animal Care and Use Committee.

### Fly infection assays

A *Drosophila melanogaster* pricking assay using *P. aeruginosa* was employed as described by Apidianakis et al. [20]. Each inoculum included approximately 100 bacteria and the assay was carried out at 21°C. The *Drosophila* feeding assay using *V. cholerae* was adapted from a previous protocol [38] in which 5 ml of 1∶50 water-diluted LB (Fisher Scientific) culture (original OD_600nm_ = 3, final CFU = 10^8^/ml) supplemented with 1% sucrose was used for each replicate. The *Drosophila* feeding assay used to test *Y. pseudotuberculosis* virulence was developed specifically for this study where 5 ml of 1∶1.25 water-diluted LB culture (original OD_600nm_ = 2, final CFU  = 10^9^/ml) supplemented with 4% sucrose was used. Five to nine day-old female flies were incubated at 29°C in both feeding assays. Fly survival was monitored until 100% mortality was achieved.

### Plant infection assay

About 10^5^ bacterial cells/leaf were forced into the intercellular space of 5-week old *Arabidopsis thaliana* ecotype Llagostera (Ll-O) leaves [13]. Throughout the course of the infection experiment, the plants were kept in a growth chamber at 30°C with a high relative humidity 80–90%. For each bacterial strain, cells were recovered from 4 different leaves; two samples were collected from each specimen leaf. CFUs were counted at days 0, 2 and 4 post-infection by selecting on rifampicin (100 µg/ml) LB agar plates.

### Amoeba phagocytosis assay

In the *D. discoideum* phagocytosis model [21], nine dilutions of *Dictyostelium* cells with 2.5-fold interval, starting from 2.0×10^5^ cells, were spotted onto bacterial lawns formed by the tested *P. aeruginosa* strains. After 5 days of incubation at 25°C, the numbers of clear zones in the lawns were recorded.

### Mouse acute lung infection assay

An acute lung infection model was used as described previously [22]. Briefly, *P. aeruginosa* cells were grown to OD_600nm_ = 3, harvested, and washed in saline. Twenty-microliter aliquots of bacterial solution containing 10^7^ bacteria were administered intranasally to each mouse (N = 8 mice/experimental group). Mouse survival was monitored for 4 days.

### Burn-mouse infection model

The assay followed a previously published protocol with modifications [23]. Briefly, after mouse anesthetization, a full-thickness thermal burn injury was produced, which involved 5%–8% of the body surface area, on the dermis of the shaved abdomen. Subsequently an inoculum of 1×10^5^ *P. aeruginosa* cells was injected intradermally into the burn eschar. Mice survival was monitored for 5 days. Experiments were repeated twice with 8 mice/bacterial group for each set.

### Infection assay statistical analysis

All of the infection assays were carried out at least 3 times unless otherwise noted. Each figure shows one representative dataset for each assay except Figure 1B, Figure 4 and Figure S2, which present compilations of multiple datasets. Survival curves were analyzed by Kaplan-Meier logrank test using MedCalc; CFUs were analyzed by student's t-tests and error bars represent standard deviation in one dataset. A P-value below 0.05 was considered significant in all cases.

### Bacterial mutant construction

The *P.a.-kerV* mutant was made in PA14 background with the first 2 and last 7 codons intact, separated by a 6-nucleotide KpnI sequence, using a modified SOE-PCR protocol [45] and pEX18Ap [46]. Complementation of the *P.a.-kerV* mutants was achieved by replacing the mutated locus with the wild-type locus [46]. *V.c.-kerV* mutants were derived from a transposon library using a clinical isolate belonging to the O1 El Tor biotype as the wild-type [37]. The three *V. cholerae kerV* mutants, *V.c.-kerV1* (EC1860), *V.c.-kerV2* (EC14225) and *V.c.-kerV3* (EC5287), had the transposon inserted into the gene ortholog at position 17.6%, 32.8% and 16.9%, respectively. The transposon and the gene were in the same orientation in all cases. *Y.p.-kerV* was constructed to have a kanamycin cassette flanked by the first 21 and last 5 amino acids of YPK_1107 in virulent YPIII (pIB1^+^) background [47].

### PA14 gene expression profiling

PA14 was grown in LB broth at 37°C to OD_600nm_ = 1, 2, 3 and 4. At each time point, cell aliquots were collected and processed for mRNA extraction, cDNA synthesis, and hybridization as described by Déziel et al. [48]. Data presented were from one experiment where three replicates from cultures at OD_600nm_ = 1, 2 and 3 and two replicates from cultures at OD_600nm_ = 4 were collected. The data were normalized using GeneSpring software (Agilent Technologies).

### Bacterial growth curves

Growth curves were performed using Sunrise^TM^ microplate absorbance reader (Tecan) in triplicates at 37°C with shaking mode set at “Normal”. Each curve was started by diluting overnight culture 1∶100 in 200 µl media. LB broth was used as rich medium; M9 minimal salts (Sigma) supplemented with 0.4% glucose was used as minimal medium.

## Supporting Information

Table S1 Raw data from Dictyostelium phagocytosis experiments.
(0.01 MB PDF)

Table S2 List of P. aeruginosa kerV and its 196 orthologs analyzed in this manuscript.
(0.34 MB DOC)

Figure S1 PA14, P.a.-kerV and P.a.-kerV-C growth curves in rich and minimal media.
(1.33 MB TIF)

Figure S2 P.a.-kerV mutant exhibits similar virulence phenotype as the parental strain in a burn-mouse model.
(0.70 MB TIF)

Figure S3 Alignment of methyltransferase type_11 motifs in PA14 kerV gene and Pfam08241.
(0.24 MB TIF)
