# Supplementary material for: The Pathogenic Properties of a Novel and Conserved Gene Product, KerV, in Proteobacteria
Source: PLoS One. 2009 Sep 25;4(9):e7167. doi: 10.1371/journal.pone.0007167 (PMC2744870; doi:10.1371/journal.pone.0007167)
Supplement: Table S1 — Raw data from Dictyostelium phagocytosis experiments. (0.01 MB PDF) [file pone.0007167.s001.pdf]

Table S1. Raw data from *Dictyostelium* phagocytosis experiments.

**Number of clear zones**	Exp. 1 Rep. 1	Exp. 1 Rep. 2	Exp. 1 Rep. 3	Exp. 2 Rep. 1	Exp. 2 Rep. 2	Exp. 2 Rep. 3	Exp. 3 Rep. 1	Exp. 3 Rep. 2	Exp. 3 Rep. 3
PA14	1	1	1	1	2	1	1	2	1
P.a.-kerV	5	5	5	5	5	5	5	5	5
P.a.-kerV-C	2	2	2	2	2	2	2	2	2
lasR	8	8	8	8	9	8	8	8	8

Note: Experiments 1 and 2 were carried out with *Dictyostelium discoideum* strain AX4 and Experiment 3 with strain DH1-10.
