# Supplementary material for: The Pathogenic Properties of a Novel and Conserved Gene Product, KerV, in Proteobacteria
Source: PLoS One. 2009 Sep 25;4(9):e7167. doi: 10.1371/journal.pone.0007167 (PMC2744870; doi:10.1371/journal.pone.0007167)
Supplement: Table S2 — List of P. aeruginosa kerV and its 196 orthologs analyzed in this manuscript. (0.34 MB DOC) [file pone.0007167.s002.doc]

**Table S2 List of *P. aeruginosa kerV* and its 196 orthologs analyzed in this manuscript.**

| **Number** | **KEEG Entry** | **Species Name** | **Conserved *gloB* Position?** | **Conserved *rnhA* Position?** | **Conserved *dnaQ* Position?** | **Gene Name** | **Length (amino acid)** | **SW-score** | **Identity** | |
| --- | --- | --- | --- | --- | --- | --- | --- | --- | --- | --- |
| **1** | pau | *Pseudomonas aeruginosa PA14* | Y | Y | Y | *kerV* | 253 | \ | \ | |
| **2** | pae | *Pseudomonas aeruginosa PAO1* | Y | Y | Y | PA1814 hypothetical protein | 253 | 1789 | 1.000 | |
| **3** | pap | *Pseudomonas aeruginosa PA7* | Y | Y | Y | PSPA7_3482 hypothetical protein | 253 | 1779 | 0.984 | |
| **4** | pmy | *Pseudomonas mendocina* | Y | Y | Y | Pmen_2065 hypothetical protein | 252 | 1357 | 0.768 | |
| **5** | psb | *Pseudomonas syringae pv. syringae B728a* | Y | Y | Y | Psyr_1762 hypothetical protein | 253 | 1327 | 0.770 | |
| **6** | pfo | *Pseudomonas fluorescens PfO-1* | Y | Y | Y | Pfl_2185 hypothetical protein | 252 | 1325 | 0.738 | |
| **7** | pst | *Pseudomonas syringae pv. tomato DC3000* | Y | Y | Y | PSPTO_3713 hypothetical protein | 253 | 1319 | 0.766 | |
| **8** | pfl | *Pseudomonas fluorescens Pf-5* | Y | Y |  | PFL_3298 hypothetical protein | 252 | 1318 | 0.730 | |
| **9** | psp | *Pseudomonas syringae pv. phaseolicola 1448A* | Y | Y |  | PSPPH_1711 hypothetical protein | 253 | 1292 | 0.754 | |
| **10** | pen | *Pseudomonas entomophila* | Y | Y | Y | PSEEN3560 hypothetical protein | 259 | 1286 | 0.733 | |
| **11** | ppf | *Pseudomonas putida F1* | Y | Y | Y | Pput_1722 hypothetical protein | 257 | 1278 | 0.725 | |
| **12** | ppu | *Pseudomonas putida KT2440* | Y | Y | Y | PP_4143 hypothetical protein | 266 | 1278 | 0.725 | |
| **13** | psa | *Pseudomonas stutzeri* | Y | Y | Y | PST_2253 SAM-dependent methyltransferase | 256 | 1159 | 0.659 | |
| **14** | hch | *Hahella chejuensis* | Y | Y | Y | HCH_02526 SAM-dependent methyltransferase | 259 | 476 | 0.370 | |
| **15** | tbd | *Thiobacillus denitrificans* | Y | Y | Y | Tbd_1664 methyl-transferase | 245 | 433 | 0.342 | |
| **16** | bpd | *Burkholderia pseudomallei 668* | Y | Y | Y | BURPS668_1467 hypothetical protein | 270 | 406 | 0.332 | |
| **17** | bpl | *Burkholderia pseudomallei 1106a* | Y | Y | Y | BURPS1106A_1497 hypothetical protein | 270 | 406 | 0.332 | |
| **18** | bmn | *Burkholderia mallei NCTC 10247* | Y | Y | Y | BMA10247_0557 hypothetical protein | 270 | 406 | 0.332 | |
| **19** | bml | *Burkholderia mallei NCTC 10229* | Y | Y | Y | BMA10299_A0595 hypothetical protein | 270 | 406 | 0.332 | |
| **20** | bmv | *Burkholderia mallei SAVP1* | Y | Y | Y | BMASAVP1_A1274 hypothetical protein | 270 | 406 | 0.332 | |
| **21** | bpm | *Burkholderia pseudomallei 1710b* | Y | Y | Y | BURPS1710b_1601 hypothetical protein | 270 | 406 | 0.332 | |
| **22** | bma | *Burkholderia mallei ATCC 23344* | Y | Y | Y | BMA0764 hypothetical protein | 270 | 406 | 0.332 | |
| **23** | bps | *Burkholderia pseudomallei K96243* | Y | Y | Y | BPSL1343 hypothetical protein | 270 | 406 | 0.332 | |
| **24** | bte | *Burkholderia thailandensis* | Y | Y | Y | BTH_I2789 hypothetical protein | 270 | 405 | 0.328 | |
| **25** | bam | *Burkholderia cepacia* | Y | Y | Y | Bamb_1163 methyltransferase type 11 | 270 | 400 | 0.344 | |
| **26** | abo | *Alcanivorax borkumensis* | Y | Y | Y | ABO_1223 hypothetical protein | 258 | 398 | 0.333 | |
| **27** | bxe | *Burkholderia xenovorans* | Y | Y | Y | Bxe_A1252 hypothetical protein | 289 | 398 | 0.382 | |
| **28** | bpa | *Bordetella parapertussis* | Y | Y |  | BPP3835 hypothetical protein | 256 | 398 | 0.315 | |
| **29** | bbr | *Bordetella bronchiseptica* | Y | Y |  | BB4279 hypothetical protein | 256 | 398 | 0.315 | |
| **30** | bpe | *Bordetella pertussis* | Y | Y |  | BP3212 hypothetical protein | 256 | 396 | 0.315 | |
| **31** | bch | *Burkholderia cenocepacia HI2424* | Y | Y | Y | Bcen2424_1286 methyltransferase type 11 | 270 | 393 | 0.397 | |
| **32** | bcn | *Burkholderia cenocepacia AU1054* | Y | Y | Y | Bcen_0805 methyltransferase type 11 | 270 | 393 | 0.397 | |
| **33** | mfa | *Methylobacillus flagellatus* | Y | Y | Y | Mfla_1480 methyltransferase type 11 | 240 | 393 | 0.307 | |
| **34** | eba | *Azoarcus sp. EbN1* |  | Y |  | ebA6459 generic methyl-transferase | 246 | 393 | 0.329 | |
| **35** | mpt | *Methylibium petroleiphilum* | Y | Y | Y | Mpe_A2528 hypothetical protein | 281 | 392 | 0.324 | |
| **36** | nmu | *Nitrosospira multiformis* | Y | Y | Y | Nmul_A1620 generic methyl-transferase | 259 | 392 | 0.310 | |
| **37** | bmu | *Burkholderia multivorans* | Y | Y | Y | Bmul_2034 methyltransferase type 11 | 270 | 390 | 0.328 | |
| **38** | bvi | *Burkholderia vietnamiensis* | Y | Y | Y | Bcep1808_1239 methyltransferase type 11 | 270 | 389 | 0.409 | |
| **39** | bur | *Burkholderia sp. 383* | Y | Y | Y | Bcep18194_A4428 hypothetical protein | 270 | 389 | 0.395 | |
| **40** | pna | *Polaromonas naphthalenivorans* | Y | Y |  | Pnap_1679 methyltransferase type 11 | 273 | 379 | 0.313 | |
| **41** | rfr | *Rhodoferax ferrireducens* | Y | Y |  | Rfer_1463 hypothetical protein | 233 | 377 | 0.332 | |
| **42** | rme | *Ralstonia metallidurans* | Y | Y | Y | Rmet_2207 methyltransferase type 11 | 264 | 376 | 0.311 | |
| **43** | rso | *Ralstonia solanacearum* | Y | Y |  | RSc1514 hypothetical protein | 257 | 375 | 0.350 | |
| **44** | neu | *Nitrosomonas europaea* | Y | Y | Y | NE0139 generic methyl-transferase | 278 | 373 | 0.309 | |
| **45** | ajs | *Acidovorax sp. JS42* | Y | Y |  | Ajs_1744 methyltransferase type 11 | 259 | 371 | 0.317 | |
| **46** | noc | *Nitrosococcus oceani* |  | Y |  | Noc_2816 generic methyl-transferase | 256 | 368 | 0.322 | |
| **47** | pol | *Polaromonas sp. JS666* |  | Y |  | Bpro_2280 hypothetical protein | 278 | 366 | 0.308 | |
| **48** | dar | *Dechloromonas aromatica* | Y | Y | Y | Daro_1594 generic methyl-transferase | 238 | 359 | 0.307 | |
| **49** | pnu | *Polynucleobacter sp. QLW-P1DMWA-1* | Y | Y |  | Pnuc_1023 methyltransferase type 11 | 272 | 353 | 0.303 | |
| **50** | pha | *Pseudoalteromonas haloplanktis* | Y | Y | Y | PSHAa1965 S-adenosyl-L-methionine-dependent methylt | 255 | 353 | 0.323 | |
| **51** | net | *Nitrosomonas eutropha* | Y | Y | Y | Neut_2177 generic methyl-transferase | 235 | 349 | 0.295 | |
| **52** | cvi | *Chromobacterium violaceum* | Y | Y | Y | CV_1255 hypothetical protein | 254 | 347 | 0.314 | |
| **53** | reh | *Ralstonia eutropha H16* | Y | Y | Y | H16_A2465 methyltransferase | 243 | 344 | 0.354 | |
| **54** | vei | *Verminephrobacter eiseniae* | Y | Y |  | Veis_0592 methyltransferase type 11 | 274 | 343 | 0.345 | |
| **55** | azo | *Azoarcus sp. BH72* |  | Y |  | azo2056 hypothetical protein | 254 | 340 | 0.305 | |
| **56** | aav | *Acidovorax avenae* | Y | Y |  | Aave_2652 methyltransferase type 11 | 251 | 339 | 0.355 | |
| **57** | sde | *Saccharophagus degradans* | Y |  | Y | Sde_2022 generic methyl-transferase | 294 | 333 | 0.301 | |
| **58** | maq | *Marinobacter aquaeolei* | Y | Y | Y | Maqu_1535 methyltransferase type 11 | 269 | 327 | 0.311 | |
| **59** | har | *Herminiimonas arsenicoxydans* | Y | Y | Y | HEAR2320 hypothetical protein | 259 | 322 | 0.340 | |
| **60** | cps | *Colwellia psychrerythraea* | Y |  |  | CPS_2000 hypothetical protein | 257 | 312 | 0.293 | |
| **6**1 | aha | *Aeromonas hydrophila* |  | Y |  | AHA_1569 methyltransferase | 244 | 311 | 0.329 | |
| **62** | sdn | *Shewanella denitrificans* | Y | Y | Y | Sden_2020 methyltransferase type 11 | 239 | 311 | 0.292 | |
| **63** | sbn | *Shewanella baltica OS195* | Y |  |  | Sbal195_2060 methyltransferase type 11 | 245 | 307 | 0.298 | |
| **64** | sbm | *Shewanella baltica OS185* | Y |  |  | Shew185_2012 methyltransferase type 11 | 245 | 307 | 0.298 | |
| **65** | sbl | *Shewanella baltica OS155* | Y |  |  | Sbal_1997 methyltransferase type 11 | 245 | 307 | 0.298 | |
| **66** | asa | *Aeromonas salmonicida* | Y | Y | Y | ASA_2789 methyltransferase | 240 | 300 | 0.329 | |
| **67** | son | *Shewanella oneidensis* | Y |  |  | SO_2562 hypothetical protein | 244 | 300 | 0.286 | |
| **68** | cbs | *Coxiella burnetii RSA 331* | Y | Y |  | COXBURSA331_A0423 putative methyltransferase | 243 | 299 | 0.314 | |
| **69** | cbu | *Coxiella burnetii RSA 493* | Y | Y | Y | CBU_0315 hypothetical protein | 243 | 299 | 0.314 | |
| **70** | cbd | *Coxiella burnetii Dugway 5J108-111* | Y | Y |  | COXBU7E912_1764 putative methyltransferase | 243 | 297 | 0.314 | |
| **71** | pin | *Psychromonas ingrahamii* | Y | Y | Y | Ping_0497 methyltransferase type 11 | 246 | 296 | 0.277 | |
| **72** | shm | *Shewanella sp. MR-7* | Y |  |  | Shewmr7_2237 methyltransferase type 11 | 240 | 295 | 0.281 | |
| **73** | she | *Shewanella sp. MR-4* | Y |  |  | Shewmr4_2160 methyltransferase type 11 | 244 | 295 | 0.281 | |
| **74** | shn | *Shewanella sp. ANA-3* | Y |  |  | Shewana3_2368 methyltransferase type 11 | 244 | 292 | 0.286 | |
| **75** | pat | *Pseudoalteromonas atlantica* | Y |  |  | Patl_1896 hypothetical protein | 249 | 291 | 0.320 | |
| 76 | ngo | *Neisseria gonorrhoeae* |  |  |  | NGO0609 hypothetical protein | 221 | 291 | 0.374 | |
| 77 | nmn | *Neisseria meningitidis 053442* |  |  |  | NMCC_1209 hypothetical protein | 205 | 290 | 0.335 | |
| 78 | nmc | *Neisseria meningitidis FAM18* |  |  |  | NMC1233 hypothetical protein | 221 | 290 | 0.361 | |
| 79 | nma | *Neisseria meningitidis Z2491 (serogroup A)* |  |  |  | NMA1506 hypothetical protein | 221 | 290 | 0.361 | |
| **80** | ecs | *Escherichia coli O157 Sakai (EHEC)* | Y | Y | Y | ECs0209 hypothetical protein | 246 | 287 | 0.317 | |
| **81** | ece | *Escherichia coli O157 EDL933 (EHEC)* | Y |  |  | Z0237 hypothetical protein | 246 | 287 | 0.317 | |
| 82 | nme | *Neisseria meningitidis MC58 (serogroup B)* |  |  |  | NMB1296 hypothetical protein | 221 | 285 | 0.361 | |
| **83** | vfi | *Vibrio fischeri* | Y | Y | Y | VF1937 methyltransferase | 247 | 284 | 0.290 | |
| **84** | ecv | *Escherichia coli APEC O1* | Y | Y | Y | APECO1_1777 putative S-adenosyl-L-methionine-depend | 246 | 281 | 0.312 | |
| **85** | ecp | *Escherichia coli 536 (UPEC)* | Y | Y | Y | ECP_0219 hypothetical protein YafS | 241 | 281 | 0.312 | |
| **86** | eci | *Escherichia coli UTI89 (UPEC)* | Y | Y | Y | UTI89_C0232 hypothetical protein YafS | 246 | 281 | 0.312 | |
| **87** | ecc | *Escherichia coli CFT073 (UPEC)* | Y | Y | Y | c0250 hypothetical protein YafS | 246 | 281 | 0.312 | |
| **88** | sfv | *Shigella flexneri 8401 (serotype 5b)* | Y |  |  | SFV_0197 hypothetical protein | 240 | 280 | 0.308 | |
| **89** | sbo | *Shigella boydii* | Y | Y | Y | SBO_0202 hypothetical protein | 241 | 280 | 0.308 | |
| **90** | sfx | *Shigella flexneri 2457T (serotype 2a)* | Y | Y | Y | S0207 hypothetical protein | 241 | 280 | 0.308 | |
| **91** | sfl | *Shigella flexneri 301 (serotype 2a)* | Y |  |  | SF0199 hypothetical protein | 241 | 280 | 0.308 | |
| **92** | eco | *Escherichia coli K-12 MG1655* | Y | Y | Y | b0213 predicted S-adenosyl-L-methionine-dependent m | 240 | 278 | 0.308 | |
| **93** | ecj | *Escherichia coli K-12 W3110* | Y | Y | Y | JW0203 predicted S-adenosyl-L-methionine-dependent | 240 | 278 | 0.308 | |
| **94** | sdy | *Shigella dysenteriae* | Y | Y | Y | SDY_0232 hypothetical protein | 246 | 277 | 0.308 | |
| **95** | ppr | *Photobacterium profundum* | Y | Y | Y | PBPRA2919 hypothetical SAM-dependent methyltransfer | 247 | 277 | 0.269 | |
| **96** | ssn | *Shigella sonnei* | Y | Y | Y | SSON_0227 hypothetical protein | 246 | 276 | 0.308 | |
| **97** | vpa | *Vibrio parahaemolyticus* | Y | Y | Y | VP2294 putative SAM-dependent methyltransferase | 251 | 275 | 0.288 | |
| **98** | aeh | *Alkalilimnicola ehrlichei* | Y | Y | Y | Mlg_1993 methyltransferase type 11 | 271 | 266 | 0.285 | |
| **99** | vvy | *Vibrio vulnificus YJ016* | Y | Y | Y | VV2531 SAM-dependent methyltransferase | 255 | 265 | 0.293 | |
| **100** | sse | *Shewanella sediminis* | Y |  |  | Ssed_1990 hypothetical protein | 244 | 262 | 0.289 | |
| **101** | shw | *Shewanella sp. W3-18-1* | Y |  |  | Sputw3181_2248 methyltransferase type 11 | 244 | 260 | 0.284 | |
| **102** | vco | *Vibrio cholerae O395* | Y | Y | Y | VC0395_A1827 hypothetical protein | 245 | 259 | 0.279 | |
| **103** | spc | *Shewanella putrefaciens* | Y |  |  | Sputcn32_1777 methyltransferase type 11 | 244 | 259 | 0.284 | |
| **104** | vch | *Vibrio cholerae O1* | Y | Y | Y | VC2235 hypothetical protein | 245 | 259 | 0.279 | |
| **105** | spt | *Salmonella enterica serovar Paratyphi A* | Y | Y | Y | SPA2507 hypothetical protein | 240 | 257 | 0.296 | |
| **106** | spl | *Shewanella pealeana* | Y |  |  | Spea_2405 methyltransferase type 11 | 246 | 256 | 0.279 | |
| **107** | slo | *Shewanella loihica* | Y | Y | Y | Shew_2113 hypothetical protein | 240 | 255 | 0.298 | |
| **108** | sec | *Salmonella enterica serovar Choleraesuis* | Y | Y | Y | SC0258 putative SAM-dependent methyltransferase | 240 | 253 | 0.292 | |
| **109** | stm | *Salmonella typhimurium LT2* | Y | Y | Y | STM0262 putative SAM-dependent methyltransferase | 240 | 253 | 0.292 | |
| **110** | spq | *Salmonella enterica serovar Paratyphi B* | Y | Y | Y | SPAB_03352 hypothetical protein | 240 | 252 | 0.296 | |
| **111** | stt | *Salmonella enterica serovar Typhi Ty2* | Y | Y | Y | t2603 hypothetical protein | 240 | 252 | 0.292 | |
| **112** | sty | *Salmonella enterica serovar Typhi CT18* | Y | Y | Y | STY0283 hypothetical protein | 240 | 252 | 0.292 | |
| **113** | ses | *Salmonella enterica subsp. Arizonae* | Y | Y | Y | SARI_02740 hypothetical protein | 240 | 251 | 0.296 | |
| **114** | ent | *Enterobacter sp. 638* | Y | Y | Y | Ent638_0747 methyltransferase type 11 | 238 | 251 | 0.288 | |
| **115** | hha | *Halorhodospira halophila* | Y | Y |  | Hhal_1586 methyltransferase type 11 | 243 | 249 | 0.289 | |
| **116** | saz | *Shewanella amazonensis* | Y |  |  | Sama_1883 hypothetical protein | 240 | 249 | 0.298 | |
| **117** | acr | *Acidiphilium cryptum JF-5* | Y |  |  | Acry_1777 methyltransferase type 11 | 235 | 243 | 0.306 | |
| **118** | ilo | *Idiomarina loihiensis* | Y |  |  | IL1696 SAM-dependent methyltransferase | 256 | 236 | 0.277 | |
| **119** | ypg | *Yersinia pestis Angola* | Y | Y | Y | YpAngola_A2684 putative methyltransferase | 239 | 228 | 0.267 | |
| **120** | ypi | *Yersinia pseudotuberculosis IP31758* | Y | Y | Y | YpsIP31758_1053 putative methyltransferase | 239 | 228 | 0.267 | |
| **121** | ypp | *Yersinia pestis Pestoides* | Y | Y | Y | YPDSF_1632 hypothetical protein | 239 | 228 | 0.267 | |
| **122** | sfr | *Shewanella frigidimarina* | Y | Y | Y | Sfri_2206 methyltransferase type 11 | 259 | 228 | 0.276 | |
| **123** | ypn | *Yersinia pestis Nepal516* | Y | Y | Y | YPN_2918 hypothetical protein | 239 | 228 | 0.267 | |
| **124** | ypa | *Yersinia pestis Antiqua* | Y | Y | Y | YPA_0558 hypothetical protein | 239 | 228 | 0.267 | |
| **125** | yps | *Yersinia pseudotuberculosis IP32953* | Y | Y | Y | YPTB2966 hypothetical protein | 239 | 228 | 0.267 | |
| **126** | ypm | *Yersinia pestis Mediaevails* | Y | Y | Y | YP_2769 SAM-dependent methyltransferase | 239 | 228 | 0.267 | |
| **127** | ypk | *Yersinia pestis KIM* | Y | Y | Y | y3096 hypothetical protein | 239 | 228 | 0.267 | |
| **128** | ype | *Yersinia pestis CO92* | Y | Y | Y | YPO1080 hypothetical protein | 239 | 228 | 0.267 | |
| **129** | kpn | *Klebsiella pneumoniae* | Y | Y | Y | KPN_00228 putative methyltransferase | 222 | 226 | 0.285 | |
| 130 | dsh | *Dinoroseobacter shibae* |  |  |  | Dshi_2938 hypothetical protein | 256 | 225 | 0.290 | |
| **131** | plu | *Photorhabdus luminescens* | Y | Y | Y | plu0941 hypothetical protein | 237 | 225 | 0.269 | |
| **132** | jan | *Jannaschia sp. CCS1* | Y |  |  | Jann_1043 hypothetical protein | 253 | 223 | 0.254 | |
| **133** | spe | *Serratia proteamaculans* | Y | Y | Y | Spro_0910 methyltransferase type 11 | 239 | 221 | 0.261 | |
| **134** | sgl | *Sodalis glossinidius* | Y | Y | Y | SG0590 hypothetical protein | 242 | 220 | 0.267 | |
| **135** | tcx | *Thiomicrospira crunogena* | Y | Y |  | Tcr_0927 hypothetical protein | 261 | 219 | 0.270 | |
| **136** | esa | *Enterobacter sakazakii* | Y | Y | Y | ESA_03126 hypothetical protein | 238 | 217 | 0.294 | |
| **137** | csa | *Chromohalobacter salexigens* | Y | Y | Y | Csal_1943 methyltransferase type 11 | 266 | 217 | 0.255 | |
| **138** | yen | *Yersinia enterocolitica* | Y | Y | Y | YE0920 hypothetical protein | 239 | 216 | 0.275 | |
| **139** | rsh | *Rhodobacter sphaeroides ATCC 17029* | Y |  |  | Rsph17029_0970 hypothetical protein | 262 | 211 | 0.280 | |
| **140** | rsp | *Rhodobacter sphaeroides 2.4.1* | Y |  |  | RSP_2295 hypothetical protein | 262 | 211 | 0.280 | |
| 141 | lpn | *Legionella pneumophila Philadelphia 1* |  |  |  | lpg1521 generic methyl-transferase | 247 | 207 | 0.249 | |
| **142** | vvu | *Vibrio vulnificus CMCP6* | Y |  |  | VV1_1884 SAM-dependent methyltransferase | 191 | 205 | 0.302 | |
| **143** | rpb | *Rhodopseudomonas palustris HaA2* | Y |  |  | RPB_0070 hypothetical protein | 254 | 204 | 0.335 | |
| **144** | rsq | *Rhodobacter sphaeroides ATCC 17025* | Y |  |  | Rsph17025_2205 hypothetical protein | 262 | 203 | 0.265 | |
| 145 | lpf | *Legionella pneumophila Lens* |  |  |  | lpl1505 hypothetical protein | 247 | 202 | 0.239 | |
| **146** | eca | *Erwinia carotovora* | Y | Y | Y | ECA3341 hypothetical protein | 236 | 199 | 0.259 | |
| **147** | rde | *Roseobacter denitrificans* | Y |  |  | RD1_3538 hypothetical protein | 244 | 198 | 0.315 | |
| 148 | lpc | *Legionella pneumophila Corby* |  |  |  | LPC_0940 generic methyl-transferase | 246 | 196 | 0.239 | |
| 149 | lpp | *Legionella pneumophila Paris* |  |  |  | lpp1478 hypothetical protein | 247 | 196 | 0.239 | |
| **150** | msu | *Mannheimia succiniciproducens* | Y |  |  | MS0945 SmtA protein | 237 | 196 | 0.269 | |
| **151** | rpd | *Rhodopseudomonas palustris BisB5* | Y |  |  | RPD_0094 methyltransferase type 11 | 254 | 195 | 0.348 | |
| **152** | pde | *Paracoccus denitrificans* | Y |  |  | Pden_3814 methyltransferase type 11 | 260 | 194 | 0.331 | |
| **153** | rpa | *Rhodopseudomonas palustris CGA009* | Y |  |  | RPA0609 hypothetical protein | 259 | 193 | 0.329 | |
| **154** | mgm | *Magnetococcus sp. MC-1* | Y |  |  | Mmc1_1658 methyltransferase type 11 | 256 | 186 | 0.266 | |
| **155** | gox | *Gluconobacter oxydans* | Y |  |  | GOX0281 putative methyltransferase | 243 | 179 | 0.276 | |
| **156** | sit | *Silicibacter sp. TM1040* | Y |  |  | TM1040_2090 hypothetical protein | 248 | 177 | 0.278 | |
| **157** | rpe | *Rhodopseudomonas palustris BisA53* | Y |  |  | RPE_0163 methyltransferase type 11 | 255 | 175 | 0.311 | |
| **158** | pmu | *Pasteurella multocida* | Y |  |  | PM0686 hypothetical E.coli | 238 | 175 | 0.363 | |
| **159** | nwi | *Nitrobacter winogradskyi* | Y |  |  | Nwi_0374 hypothetical protein | 246 | 173 | 0.287 | |
| 160 | ttj | *Thermus thermophilus HB8* |  |  |  | TTHA0482 hypothetical protein | 212 | 171 | 0.329 | |
| **161** | bja | *Bradyrhizobium japonicum* | Y |  |  | bll0221 hypothetical protein | 246 | 169 | 0.284 | |
| **162** | nha | *Nitrobacter hamburgensis* | Y |  |  | Nham_0467 hypothetical protein | 246 | 168 | 0.286 | |
| **163** | mag | *Magnetospirillum magneticum* | Y |  |  | amb4193 SAM-dependent methyltransferase | 240 | 168 | 0.238 | |
| **164** | rpc | *Rhodopseudomonas palustris BisB18* | Y |  |  | RPC_0509 methyltransferase type 11 | 257 | 165 | 0.298 | |
| 165 | mca | *Methylococcus capsulatus* |  |  |  | MCA2029 hypothetical protein | 246 | 165 | 0.258 | |
| **166** | bbt | *Bradyrhizobium sp. BTAi1* | Y |  |  | BBta_7608 hypothetical protein | 255 | 163 | 0.263 | |
| **167** | pla | *Parvibaculum lavamentivorans* | Y |  |  | Plav_1579 methyltransferase type 11 | 252 | 160 | 0.257 | |
| **168** | gbe | *Granulobacter bethesdensis* | Y |  |  | GbCGDNIH1_0454 methyltransferase | 269 | 159 | 0.269 | |
| **169** | xom | *Xanthomonas oryzae MAFF311018* | Y | Y | Y | XOO_0939 hypothetical protein | 218 | 159 | 0.307 | |
| **170** | xoo | *Xanthomonas oryzae KACC10331* | Y | Y | Y | XOO1040 hypothetical protein | 218 | 159 | 0.307 | |
| **171** | bra | *Bradyrhizobium sp. ORS278* | Y |  |  | BRADO0568 conserved hypothetical protein; putative | 268 | 157 | 0.267 | |
| **172** | sil | *Silicibacter pomeroyi* | Y |  |  | SPO3167 hypothetical protein | 250 | 157 | 0.256 | |
| **173** | mes | *Mesorhizobium sp. BNC1* | Y |  |  | Meso_3081 methyltransferase type 11 | 258 | 156 | 0.277 | |
| **174** | mmr | *Maricaulis maris* | Y |  |  | Mmar10_2380 methyltransferase type 11 | 255 | 151 | 0.313 | |
| **175** | xcb | *Xanthomonas campestris pv. campestris 8004* | Y | Y | Y | XC_3257 hypothetical protein | 218 | 150 | 0.292 | |
| **176** | xcc | *Xanthomonas campestris pv. campestris ATCC 33913* | Y | Y | Y | XCC0986 hypothetical protein | 218 | 150 | 0.292 | |
| **177** | smd | *Sinorhizobium medicae* | Y |  |  | Smed_2551 methyltransferase type 11 | 250 | 147 | 0.256 | |
| **178** | sme | *Sinorhizobium meliloti* | Y |  |  | SMc00709 hypothetical protein | 254 | 145 | 0.260 | |
| **179** | rle | *Rhizobium leguminosarum* | Y |  |  | RL4339 hypothetical protein | 258 | 141 | 0.281 | |
| **180** | rru | *Rhodospirillum rubrum* | Y |  |  | Rru_A3269 hypothetical protein | 264 | 140 | 0.292 | |
| 181 | bme | [*Brucella melitensis*](http://www.genome.jp/dbget-bin/www_bfind?B.melitensis) |  |  |  | BMEI0081 methyltransferase | 308 | 140 | 0.276 | |
| **182** | atu | *Agrobacterium tumefaciens C58 (UWash/Dupont)* | Y |  |  | Atu3613 hypothetical protein | 253 | 140 | 0.276 | |
| **183** | atc | *Agrobacterium tumefaciens C58 (Cereon)* | Y |  |  | AGR_L_2422gl hypothetical protein | 288 | 140 | 0.276 | |
| 184 | bmt | *Brucella suis ATCC 23445* |  |  |  | BSUIS_A1826 ATP synthase subunits region ORF 4 | 259 | 139 | 0.281 | |
| **185** | ret | *Rhizobium etli* | Y |  |  | RHE_CH03811 putative SAM-dependent methyltransferas | 221 | 139 | 0.282 | |
| **186** | xcv | *Xanthomonas campestris pv. Vesicatoria* | Y | Y | Y | XCV1088 hypothetical protein | 218 | 139 | 0.292 | |
| 187 | bcs | *Brucella canis* |  |  |  | BCAN_A2031 ATP synthase subunits region ORF 4 | 259 | 138 | 0.281 | |
| 188 | bov | *Brucella ovis* |  |  |  | BOV_1911 hypothetical protein | 234 | 138 | 0.281 | |
| 189 | bmf | *Brucella melitensis biovar Abortus* |  |  |  | BAB1_1987 generic methyltransferase | 259 | 138 | 0.281 | |
| 190 | bmb | *Brucella abortus* |  |  |  | BruAb1_1962 hypothetical protein | 259 | 138 | 0.281 | |
| 191 | bms | *Brucella suis* |  |  |  | BR1986 hypothetical protein | 259 | 138 | 0.281 | |
| **192** | xac | *Xanthomonas axonopodis* | Y | Y | Y | XAC1088 hypothetical protein | 218 | 138 | 0.292 | |
| 193 | oan | *Ochrobactrum anthropi* |  |  |  | Oant_0994 methyltransferase type 11 | 259 | 133 | 0.261 | |
| 194 | bbk | *Bartonella bacilliformis* |  |  |  | BARBAKC583_0058 putative SAM-dependent methyltransf | 244 | 131 | 0.290 | |
| **195** | xft | *Xylella fastidiosa Temecula1* | Y | Y | Y | PD1219 hypothetical protein | 216 | 129 | 0.258 | |
| **196** | xfa | *Xylella fastidiosa 9a5c* | Y | Y | Y | XF2159 hypothetical protein | 216 | 129 | 0.258 | |
| **197** | hso | *Haemophilus somnus* | Y |  |  | HS_0808 possible SAM-dependent methyltransferase | 241 | 117 | 0.240 | |
|  |  |  |  |  |  |  |  |  |  |  |

Note:

1. *kerV* ortholog in strain YPIII is YPK_1107 and it is 100% identical to k*erV* ortholog YPTB2966 in strain IP32953
2. Typical configuration is: *gloB*-*kerV*-*rnhA*-*dnaQ*
3. Bold numbered species, 176 in total, contain the conserved genomic neighborhood, meaning that at least one of *gloB* and *rnhA* orthologs keeps the typical configuration regarding to *kerV* ortholog and the remaining gene orthologs are present in the genome, if not nearby.
