# Supplementary figures and images for: The Pathogenic Properties of a Novel and Conserved Gene Product, KerV, in Proteobacteria
Source: PLoS One. 2009 Sep 25;4(9):e7167. doi: 10.1371/journal.pone.0007167 (PMC2744870; doi:10.1371/journal.pone.0007167)

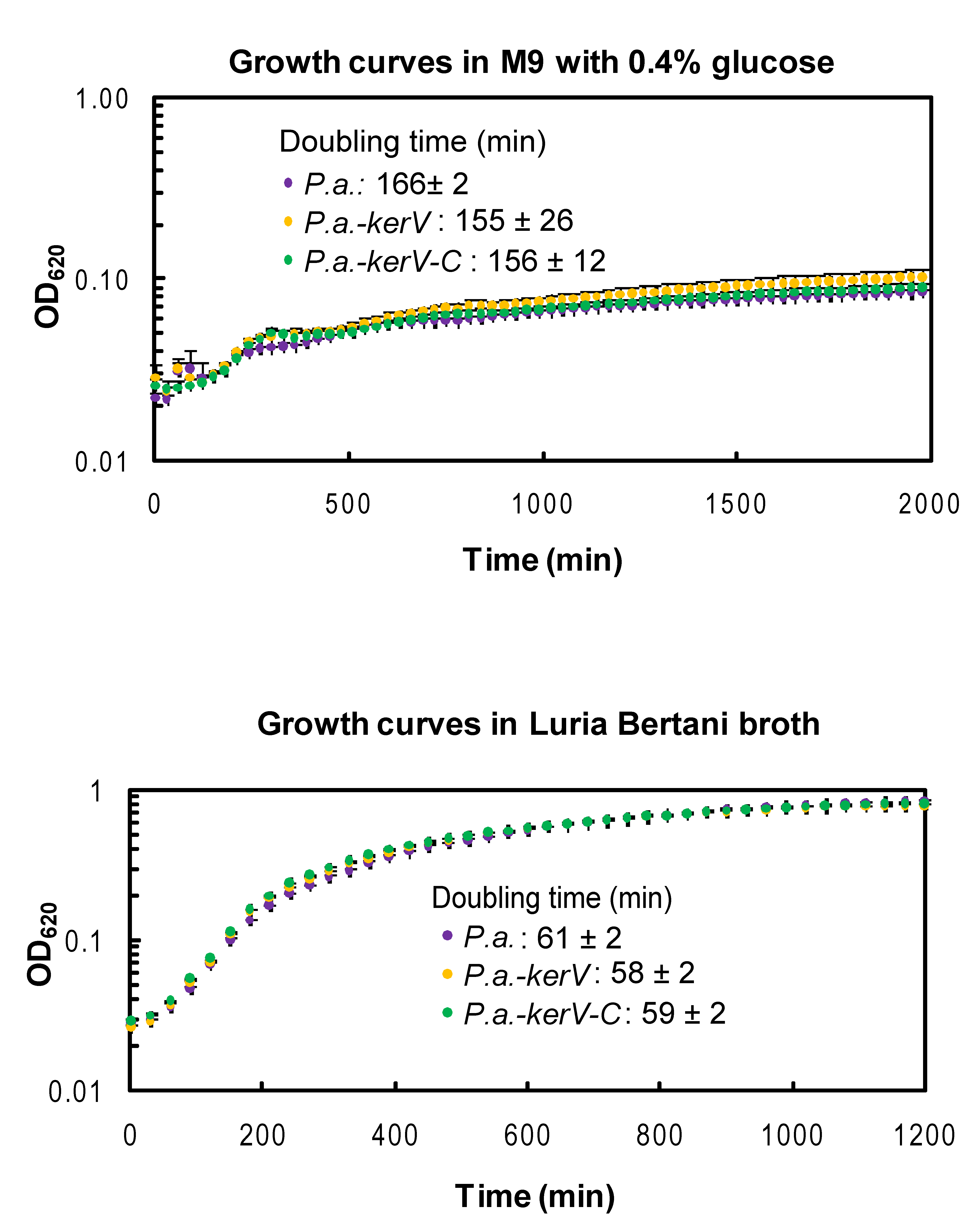

Supplement: Figure S1 — PA14, P.a.-kerV and P.a.-kerV-C growth curves in rich and minimal media. (1.33 MB TIF) [file pone.0007167.s003.tif]

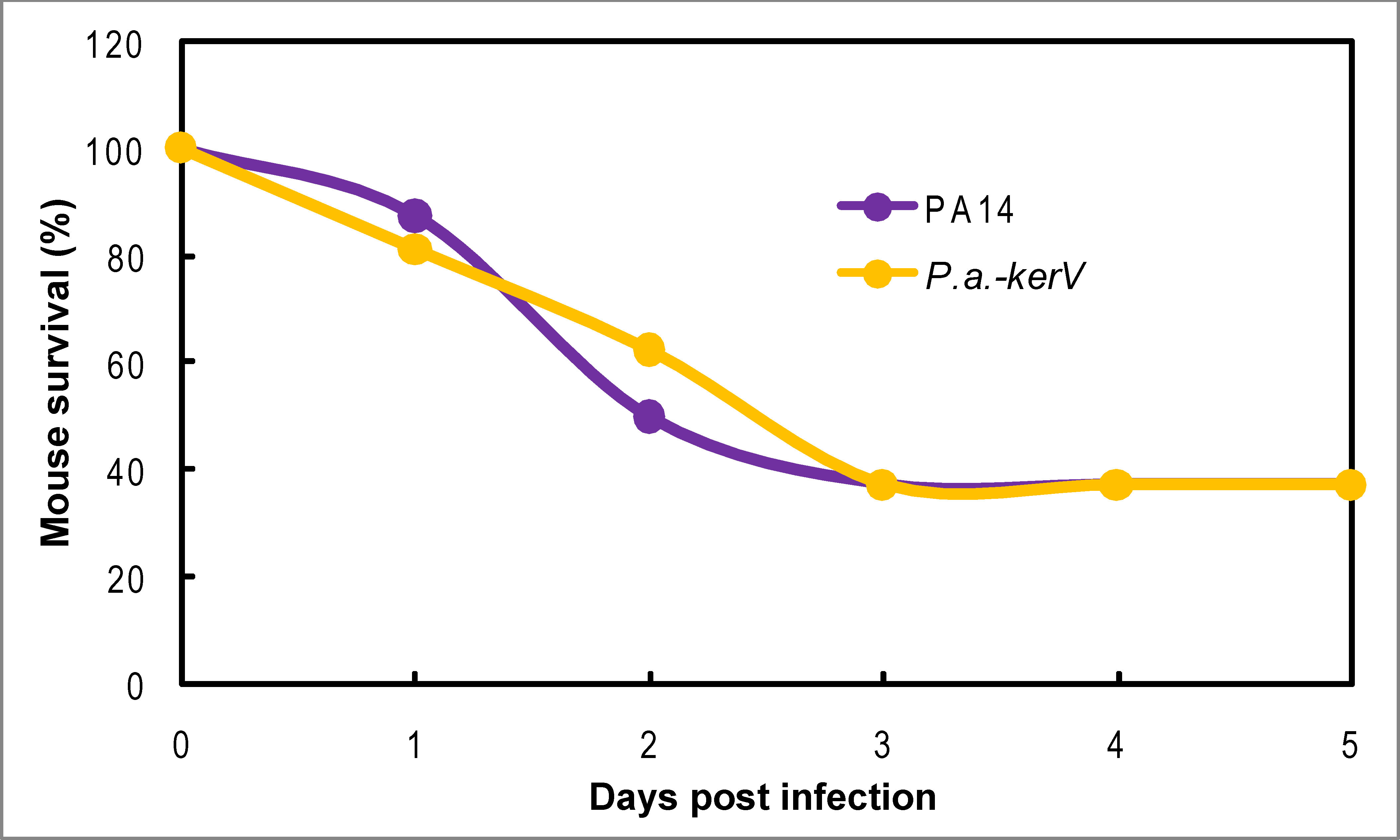

Supplement: Figure S2 — P.a.-kerV mutant exhibits similar virulence phenotype as the parental strain in a burn-mouse model. (0.70 MB TIF) [file pone.0007167.s004.tif]

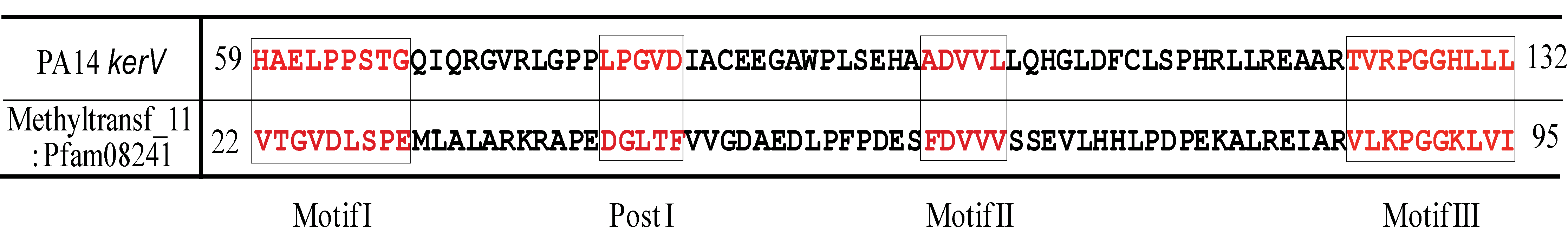

Supplement: Figure S3 — Alignment of methyltransferase type_11 motifs in PA14 kerV gene and Pfam08241. (0.24 MB TIF) [file pone.0007167.s005.tif]
